# Dual subclavian-brachiocephalic artery tortuosity and its impact on procedural efficiency in trans-radial access cerebral angiography: a retrospective cohort study

**DOI:** 10.1186/s12883-026-04799-4

**Published:** 2026-03-10

**Authors:** Qing Tian, ZhenBao Liu, YaNan Zhao, JiaChen Gu

**Affiliations:** 1 Department of Neurology, Qingpu Branch of Zhongshan Hospital, Fudan University, Shanghai, 201799 P. R. China; 2 Department of Neurology, Shanghai General Hospital, Shanghai Jiao Tong University School of Medicine, Shanghai, 200080 P. R. China

**Keywords:** Trans-radial access, Cerebral angiography, Fluoroscopy time

## Abstract

**Background:**

While trans-radial access (TRA) offers advantages in cerebral angiography, its efficiency is compromised by uncharacterized neurovascular anatomical barriers. This study investigates the impact of arterial tortuosity on procedural efficiency in TRA cerebral angiography.

**​methods:**

In a retrospective cohort of patients undergoing TRA cerebral angiography at our center (Oct 2023-Apr 2024), vascular tortuosity was classified via intraprocedural angiography. Procedural efficiency was stratified by total fluoroscopy time (Easy to Extremely Difficult grades). Multivariable ordinal regression and mediation analyses identified anatomical predictors and mechanistic pathways.

**​results:**

This study comprised 170 patients (mean age 60.66±12.62 years; 31.76% female). Procedural success was achieved in all patients. After adjusting for other confounders, dual subclavian-brachiocephalic tortuosity was found to be independent predictor of increasing procedural inefficiency. This variant prolonged total fluoroscopy time primarily through delayed left-vessel catheterization, with superselective catheterization of the left subclavian artery (SC-LSA) and superselective cannulation of the Left common carotid artery (SC-LCCA) jointly mediating 95.5%-99.2% of the total effect (parallel path coefficients a1/a2/b1/b2 all p<0.001; total indirect effect p<0.001). The contribution ratio of SC-LSA to SC-LCCA was 1.2:1 to 1.4:1, while direct effects became non-significant (c', p>0.05), confirming complete mediation. No statistical difference existed between SC-LSA and SC-LCCA's mediating effects (contrast effect c1 95% CI included zero).

**​conclusions:**

Dual subclavian-brachiocephalic tortuosity is a critical remediable obstacle to efficient neurointerventional TRA. Preprocedural screening for this variant combined with torsion-resistant catheter designs may reduce fluoroscopy time, enhancing TRA viability for cerebrovascular diagnostics.

**Supplementary Information:**

The online version contains supplementary material available at 10.1186/s12883-026-04799-4.

## Background

Cerebral angiography remains the gold standard for diagnosing cerebrovascular diseases. Currently, there are two primary clinical approaches for cerebral angiography: the trans-femoral Access (TFA) and trans-radial Access (TRA). Although TRA offers multiple advantages over TFA, including minimally invasive procedures, lower puncture site complications, no mandatory post-operative bed rest, and better patient acceptance due to reduced privacy exposure [1, 2], its clinical application faces technical challenges [3]. The relatively smaller caliber of radial arteries compared to femoral arteries increases puncture difficulty, while complex catheter looping, vessel superselection, and anatomical variations in the aortic arch and its branches often complicate the procedure [4, 5]. Notably, TRA cerebral angiography procedures demonstrate significantly prolonged fluoroscopy duration in certain patient populations [6]. Extended endovascular manipulation may substantially elevate radiation dosage and contrast medium consumption, while repeated catheter rotation maneuvers could potentially induce adverse complications such as vascular spasm, endothelial injury, catheter fatigue failure, and intraoperative embolism [7]. 

During TRA cerebral angiography, the catheter traverses sequentially through the right radial artery, right brachial artery, right axillary artery, right subclavian artery, and brachiocephalic artery before reaching the aortic arch. The anatomical morphology of these vascular structures directly determines catheter conformation and consequently impacts catheter manipulation [3]. The right subclavian-brachiocephalic arterial segment serves as the final conduit for catheter entry into the aortic arch. While the relatively large lumen diameter of this segment generally prevents significant catheter deformation, certain patients exhibit tortuous anatomical configurations in the right subclavian-brachiocephalic arteries. These morphological variations may induce catheter distortion, potentially contributing to increased technical difficulty in TRA cerebral angiography.

Therefore, this study aims to systematically evaluate how vascular anatomical morphology along the catheter pathway influences procedural complexity in *trans-right-proximal-radial-access* cerebral angiography. We hypothesize that morphology of right subclavian-brachiocephalic arterial segment significantly increases procedural complexity. The findings will guide optimization of angiographic strategies and enhance the applicability of TRA in cerebrovascular interventions.

## Methods

### Patient demographics

This retrospective cohort study, conducted in accordance with the Strengthening the Reporting of Observational Studies in Epidemiology (STROBE) guidelines [8], analyzed a prospectively maintained institutional database spanning October 2023 to April 2024. During this period, consecutive diagnostic cerebral angiography procedures were performed, with all examinations conducted in compliance with the Declaration of Helsinki. Demographic characteristics, medical comorbidities, smoking status, vascular anatomical characteristics and procedural parameters were prospectively recorded. The inclusion criteria comprised: (1) successful puncture of the right proximal radial artery and subsequent sheath placement; (2) aortic arch angiography performed using a pigtail catheter; (3) completion of superselective angiography for all four great vessels (bilateral common carotid arteries and bilateral subclavian arteries) using a 5Fr Simmons II catheter and (4) all procedures according to the *Standardized Protocol for TRA Cerebral Angiography* (as detailed below). Exclusion criteria included: (1) non-right proximal radial artery access (e.g., right distal radial artery, left radial artery, or femoral artery access); (2) crossover to an alternative vascular access after successful radial sheath placement; (3) failure to use a pigtail catheter for aortic arch angiography; (4) incomplete angiography of any of the four target great vessels; or (5) additional superselective angiography of vertebral, internal carotid, or external carotid arteries during the procedure. Ethical approval was obtained from the Institutional Review Board of Fudan University Zhongshan Hospital Qingpu Branch (NO.2023-44). The requirement for informed consent was waived due to the retrospective design and use of de-identified procedural images that posed minimal risk to participants.

### Anatomical assessment and procedural time metrics definitions​

**Fig. 1:**
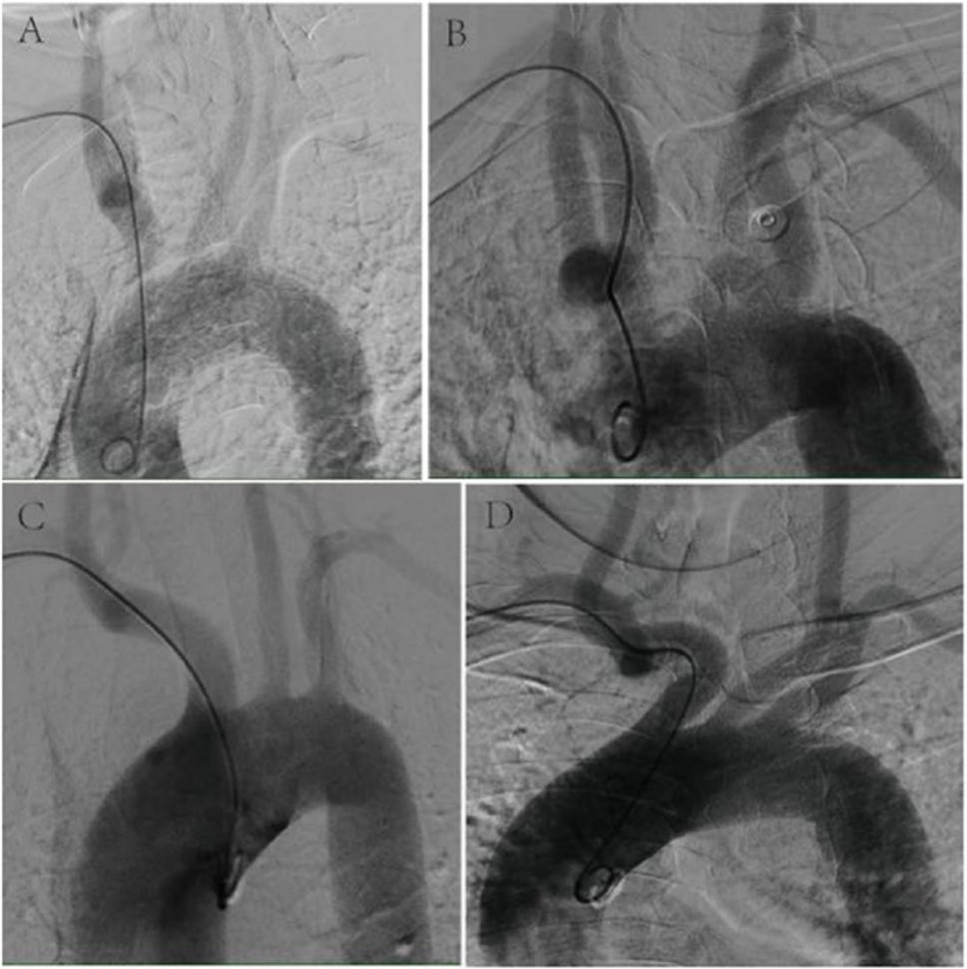
Morphology of Subclavian-brachiocephalic Artery. **A** Normal subclavian-brachiocephalic artery will not cause deformation of the catheter. **B** Single subclavian-brachiocephalic curve causes the catheter to form a kink at the junction between the subclavian artery and the innominate artery. **C** Isolated brachiocephalic curvature causes the catheter to form a mild kink within brachiocephalic artery. **D** Dual subclavian-brachiocephalic tortuosity makes the catheter to form two kinks, resulting in a "3" shape of the catheter during aortic arch angiography
- *Deformation-Based Classification*: vascular tortuosity was defined by significant catheter deformation (Tortuosity, Kinking, or Coiling) in any segment after the pigtail catheter tip was placed in the ascending aorta. The "Normal" classification constituted the absence of catheter deformation, which also included the resolution of any pre-existing vascular tortuosity through catheter support.
- *Exclusion of Physiological vascular Curve*: Due to the inherently arch-shaped anatomy inducing unavoidable tortuosity, natural catheter deformation at the subclavian-axillary artery transition was excluded.

Our study describes a streamlined assessment method that rapidly identifies anatomy affecting catheter torque by using pigtail catheter deformation within the vascular segment as the key tortuosity indicator, bypassing conventional vascular measurements for superior clinical practicality in real-time interventions. The protocol is detailed below:

- *Segmental Vascular Division:* the vascular pathway from the brachiocephalic origin to the radial artery was divided into three sequential segments (distal to proximal):
- Radial artery segment: The lumen diameter of the radial artery is comparable to catheter dimensions, permitting guidewire/catheter support to overcome most tortuous configurations and allowing direct observation of deformation.
- Brachial-axillary artery segment: the brachial and axillary arteries exhibit anatomical continuity and aligned trajectories with gradual caliber transitions, justifying their evaluation as a unified functional segment.
- Right subclavian-brachiocephalic segment: this segment was assessed as an integrated anatomical unit due to the frequent tortuosity at their junction. Based on angiographic patterns and catheter deformation, it was categorized into four types: normal, single subclavian-brachiocephalic curve, isolated brachiocephalic curvature, and dual subclavian-brachiocephalic tortuosity (Figure 1).

Aortic arch configurations followed Casserly classification (Types I-III) [9], with bovine arch defined as left common carotid origin from the brachiocephalic trunk. Two independent neurointerventional physicians performed blind evaluations of anatomical landmarks and fluoroscopy time calculations. Discrepancies were resolved through consensus review by a senior neurointerventionalist.

Procedural metrics were calculated using data from the interventional imaging system. Total fluoroscopy time was directly recorded, with radiation exposure quantified by fluoroscopy dose-area product (fDAP, μGy·m²). Phase-specific fluoroscopy durations were measured for six procedural phases: pigtail catheter positioning, Simmons catheter looping, and superselective cannulation of four arch vessels. Parameter definitions are detailed in Table 1.

**Table 1:** Description of angiographic procedures defined

Characteristics	Description
Placement time of pigtail catheter, PTPC	Total fluoroscopy time from insertion of the pigtail catheter through the radial artery sheath to its positioning in the ascending aortic angiography site.
Catheter Exchange Time, CET	Total fluoroscopy time from guidewire passage through the pigtail catheter into the descending aorta to successful loop formation of the Simmons II catheter.
Superselective Catheterization of the Left Subclavian Artery, SC-LSA	Total fluoroscopy time for superselective cannulation of the left subclavian artery
Superselective Cannulation of the Left Common Carotid Artery, SC-LCCA	Total fluoroscopy time for superselective cannulation of the Left Common Carotid Artery
superselective cannulation of the Right subclavian artery, SC-RSA	Total fluoroscopy time for superselective cannulation of the right subclavian artery
superselective cannulation of the Right Common Carotid Artery, SC-RCCA	Total fluoroscopy time for superselective cannulation of the right common carotid artery

### Standardized protocol for TRA cerebral angiography

All angiographic procedures were performed using a Philips UNIQ FD20/15 biplane DSA system with standardized “Normal” fluoroscopy mode. Patients underwent right proximal radial artery access following preprocedural evaluation of radial pulse and modified Allen test (capillary refill ≤ 10 s). Cases with absent pulse or abnormal Allen results (Barbeau Type D) were excluded and redirected to alternative access (left radial/femoral). Two fellowship-trained neurointerventional physicians (Physician 1 and Physician 2), with 6 and 4 years of independent practice experience respectively, each performed over 500 TRA cerebral angiography procedures. All surgeries were independently conducted in adherence to a predefined protocol.

Access was established using a 5 F Glidesheath Slender (Terumo) via modified Seldinger technique. A spasmolytic cocktail (verapamil 2.5 mg, nitroglycerin 200 µg, heparin 2000 IU) was administered intra-arterially. Aortic arch angiography was performed with a 5 F pigtail catheter (Cordis) over a 150-cm 0.035” hydrophilic guidewire(Terumo), utilizing 20 ml contrast at 25 ml/s in LAO 30° projection. Catheter exchange employed a 260-cm 0.035” hydrophilic guidewire (Terumo) to position a 5 F Simmons II catheter (Cordis) in the descending aorta [10], with aortic valve looping reserved for failed descending aortic shaping [11]. Superselective angiography was performed sequentially from left-sided (subclavian then common carotid arteries) to right-sided (common carotid preceding subclavian arteries) vessels. The interrogation order between ipsilateral subclavian and carotid arteries (left or right) could be adjusted intraoperatively based on technical considerations. Hemostasis was achieved with a compression band after sheath removal.

### Statistical analysis

Statistical analyses were performed using SPSS 26.0 (IBM Corp.) with the PROCESS macro (v3.5). Continuous variables were assessed for normality via Kolmogorov-Smirnov and Shapiro-Wilk tests, with parametric data expressed as mean ± SD and nonparametric data as median (interquartile range (IQR)). Group comparisons utilized ANOVA (Bonferroni post-hoc) or Kruskal-Wallis H test (Dunn’s correction) for continuous variables, and chi-square/Fisher’s exact test for categorical variables. Fluoroscopy time (right skewed) was categorized into four difficulty grades (Easy: <5 min; Moderate: 5 to < 10 min; Difficult: 10 to < 15 min; Extremely difficult: ≥15 min) through equidistant thresholding. Ordinal logistic regression identified predictors of difficulty grades, with robustness verified by multi-model comparisons and ORs (95% CIs). Mediation analysis (Baron & Kenny’s framework) evaluated anatomy’s indirect effects via procedural time using bias-corrected bootstrapping (5,000 resamples) and parallel pathway validation. Statistical significance was defined as two-tailed with a significance level set at < 0.05. This intraprocedural analysis required no follow-up, thus avoiding issues of loss to follow-up or missing data handling.

## Results

### Patient demographics​

Of 281 patients who underwent diagnostic cerebral angiography, 27 were excluded due to access-related issues. The specific reasons were: weak radial artery pulsation or abnormal Allen test results necessitating alternative access (*n* = 9), patient preference for distal radial access (*n* = 6), crossover to femoral access after radial puncture failure (*n* = 4), radial artery tortuosity preventing guidewire advancement post-sheath(*n* = 4), and anatomical constraints precluding right transradial access (absence of the right upper limb or right subclavian artery occlusion, *n* = 4). An additional 84 patients were excluded because they underwent superselective angiography, including right vertebral (*n* = 23), right internal carotid (*n* = 40), or left internal carotid (*n* = 21) artery angiography. Consequently, 170 patients met the inclusion criteria and constituted the final study cohort, stratified into four procedural difficulty grades based on total fluoroscopy time: Easy (*n* = 31), Moderate (*n* = 93), Difficult (*n* = 30), and extremely difficult (*n* = 16).

Two neurointerventional physicians independently performed the procedures (73 and 97 procedures, respectively). The distribution of procedural difficulty between the two operators’ patient groups showed no significant difference (χ²=5.85, *p* = 0.120). A comparison of procedural time metrics revealed that the difference in total fluoroscopy time between Physician 1 and Physician 2 was of borderline statistical significance (*p* = 0.05). A statistically significant difference was observed in the SC-LSA, which was 60 s for Physician 1 compared to 47 s for Physician 2 (*p* = 0.045). No significant differences were found in the remaining time parameters. Detailed comparisons are provided in the supplementary table.

Demographic analysis revealed significant intergroup differences in age (F = 5.78, *p* < 0.001) and BMI (F = 9.50, *p* < 0.001). Significant intergroup differences were observed in right subclavian-brachiocephalic anatomic configurations (*p* = 0.007). Regarding procedural parameters, statistically significant differences were observed across difficulty grades in CET (χ²=18.52, *P* < 0.001), SC-LSA (χ²=81.22, *P* < 0.001), SC-LCCA (χ²=76.72, *P* < 0.001), SC-RSA (χ²=72.01, *P* < 0.001), SC-RCCA ༈χ²=54.56, *P* < 0.001༉. All characteristics of the four groups are presented in Table 2. The results of pairwise comparisons between groups are presented in Supplementary Fig. 1 and Supplementary Fig. 2.

**Table 2:** Characteristics of patients (*n* = 170)

Characteristics	Total (*n* = 170)	Easy (*n* = 31)	Moderate (*n* = 93)	Difficult (*n* = 30)	Extremely difficult (*n* = 16)	Statistic	*P*-value
Demographics							
Age, years, Mean ± SD	61.04 ± 12.62	54.03 ± 13.23	61.04 ± 12.33	61.83 ± 11.19	69.06 ± 10.02	F = 5.78	< 0.0001*
Gender, n (%)						χ²=0.81	0.846
Male	116 (68.24)	22 (70.97)	62 (66.67)	22 (73.33)	10 (62.50)		
Female	54 (31.76)	9 (29.03)	31 (33.33)	8 (26.67)	6 (37.50)		
BMI, Mean ± SD	24.87 ± 3.35	24.56 ± 2.55	23.97 ± 2.84	27.26 ± 4.54	26.23 ± 2.39	F = 9.50	< 0.0001*
Medical history, n (%)							
Hypertension	147 (86.47)	26 (83.87)	81 (87.10)	26 (86.67)	14 (87.50)	χ²=3.09	0.379
Hyperhomocysteinemia	23 (13.53)	5 (16.13)	12 (12.90)	4 (13.33)	2 (12.50)	-	0.983
Hyperlipidaemia	70 (41.18)	14 (45.16)	34 (36.56)	16 (53.33)	6 (37.50)	χ²=2.94	0.401
Diabetes	63 (37.06)	10 (32.26)	34 (36.56)	13 (43.33)	6 (37.50)	χ²=0.82	0.844
Smoking	73 (42.94)	10 (32.26)	39 (41.94)	16 (53.33)	8 (50.00)	χ²=3.13	0.372
Anatomic Features, n (%)							
Presence of radial artery curve	15 (8.82)	1 (3.23)	6 (6.45)	4 (13.33)	4 (25.00)	-	0.053
Presence of brachial-axillary artery curve	25 (14.71)	1 (3.23)	14 (15.05)	6 (20.00)	4 (25.00)	-	0.104
Right subclavian-brachiocephalic anatomy						-	0.007*
Normal	85 (50.00)	20 (64.52)	51 (54.84)	9 (30.00)	5 (31.25)		
Presence of single subclavian-brachiocephalic curve	30 (17.65)	6 (19.35)	18 (19.35)	4 (13.33)	2 (12.50)		
Presence of isolated brachiocephalic curvature	30 (17.65)	3 (9.68)	16 (17.20)	9 (30.00)	2 (12.50)		
Presence of dual subclavian-brachiocephalic tortuosity	25 (14.71)	2 (6.45)	8 (8.60)	8 (26.67)	7 (43.75)		
Type of Aortic Arch						χ²=5.01	0.542
Type I	90 (52.94)	19 (61.29)	48 (51.61)	17 (56.67)	6 (37.50)		
Type II	47 (27.65)	6 (19.35)	26 (27.96)	10 (33.33)	5 (31.25)		
Type III	33 (19.41)	6 (19.35)	19 (20.43)	3 (10.00)	5 (31.25)		
Presence of Bovine anatomy	18 (10.59)	4 (12.90)	12 (12.90)	2 (6.67)	0 (0.00)	-	0.473
Procedural Time Metrics, second, IQR							
PTPC	34.00 (29.00–39.00)	31.00 (27.00–37.00)	33.00 (29.00–38.00)	36.50 (33.00–41.75)	35.00 (30.00–38.25)	χ²=6.38#	0.094
CET	40.00 (35.00–46.75)	37.00 (31.50–39.00)	40.00 (35.00–47.00)	43.00 (38.00–50.75)	44.50 (40.75–49.50)	χ²=18.52#	< 0.001*
SC-LSA	52.50 (33.25–85.75)	24.00 (19.50–32.00)	52.00 (35.00–72.00)	96.50 (55.50–165.75)	173.50 (69.50–237.00)	χ²=81.22#	< 0.001*
SC-LCCA	36.50 (24.00–70.00)	17.00 (13.00–25.00)	37.00 (26.00–56.00)	100.00 (56.00–167.25)	180.50 (46.75–308.75)	χ²=76.72#	< 0.001*
SC-RSA	33.00 (22.00–45.75)	19.00 (13.00–22.50)	33.00 (23.00–40.00)	40.50 (33.25–75.25)	121.50 (58.75–276.00)	χ²=72.01#	< 0.001*
SC-RCCA	30.00 (21.00–48.75)	18.00 (11.50–21.00)	31.00 (23.00–43.00)	54.50 (30.50–101.50)	68.00 (33.50–89.50)	χ²=54.56#	< 0.001*
Angiographic Features, IQR							
fDAP, Gycm²	37.61 (24.49–58.91)	19.50 (14.74–23.64)	34.00 (25.81–48.10)	67.87 (55.54–90.64)	95.38 (80.03–124.47)	χ²=97.70#	< 0.0001*
Total fluoroscopy time, min	7.27 (5.32–10.12)	4.18 (3.77–4.68)	6.78 (5.82–8.07)	11.95 (10.55–12.72)	18.32 (16.20–22.11)	χ²=139.25#	< 0.001*
Operators, n (%)						χ²=5.84	0.120
Neurointerventional physician 1	73 (42.94)	13 (41.94)	38 (40.86)	18 (60.00)	4 (25.00)		
Neurointerventional physician 2	97 (57.06)	18 (57.06)	55 (59.14)	12 (40.00)	12 (75.00)		

*BMI *body mass index, *PTPC *placement time of pigtail catheter, *CET *catheter exchange time, *SC-LSA* superselective catheterization of the left subclavian artery, *SC-LCCA* superselective cannulation of the left common carotid artery, *SC-RSA* superselective cannulation of the right subclavian artery, *SC-RCCA* superselective cannulation of the right common carotid artery, *SD *standard deviation, *IQR *interquartile range

F: ANOVA, #: Kruskal-waills test, χ²: Chi-square test, -: Fisher exact

*Statistically significant

### Anatomical factors affecting procedural efficiency in TRA cerebral angiography

Univariate ordinal logistic regression revealed that advancing age (OR 1.05 per 1-year age increase, 95%CI 1.02–1.07, *p* < 0.001), elevated BMI (OR 1.15 per 1 kg/m² increase, 95%CI 1.06–1.25, *p* < 0.001) were associated with the increasing procedural difficulty grades. Regarding anatomical features, Presence of radial artery curve (OR 3.73, 95%CI 1.37–10.19, *p* = 0.010), Presence of brachial-axillary artery curve (OR 2.41, 95%CI 1.10–5.30, *p* = 0.029), Presence of Isolated brachiocephalic curvature (Normal vs. Presence of isolated brachiocephalic curvature: OR 2.59, 95%CI 1.16–5.80, *p* = 0.021), Presence of dual subclavian-brachiocephalic tortuosity (Normal vs. Presence of dual subclavian-brachiocephalic tortuosity: OR 6.98, 95%CI 2.85–17.07, *p* < 0.001) were identified clinical predictors for increasing procedural difficulty grades. Smoking showed borderline significance (OR 1.67, 95%CI 0.93–2.99, *p* = 0.087) (Table 3).

**Table 3:** Univariate ordinal logistic regression analysis of clinical and anatomical factors associated with difficulty grades in TRA cerebral angiography

Variables	β	S. E	t-value	*P*-value	OR (95%CI)
Demographics					
Age	0.04	0.01	3.62	< 0.001*	1.05 (1.02–1.07)
Gender					
Male(Ref)					
Female	0.04	0.31	0.13	0.894	1.04 (0.56–1.93)
BMI	0.14	0.04	3.39	< 0.001*	1.15 (1.06–1.25)
Medical history					
Hypertension	0.55	0.34	1.62	0.106	1.73 (0.89–3.36)
Hyperhomocysteinemia	−0.15	0.43	−0.34	0.732	0.86 (0.37–2.01)
Hyperlipidemia	0.10	0.30	0.35	0.729	1.11 (0.62–1.99)
Diabetes	0.23	0.30	0.76	0.446	1.26 (0.70–2.28)
Smoking	0.51	0.30	1.71	0.087	1.67 (0.93–2.99)
Anatomical Features					
Presence of radial artery curve	1.32	0.51	2.57	0.010*	3.73 (1.37–10.19)
Presence of brachial-axillary artery curve	0.88	0.40	2.19	0.029*	2.41 (1.10–5.30)
Right subclavian-brachiocephalic anatomy					
Normal(Ref)					
Presence of single subclavian-brachiocephalic curve	0.21	0.42	0.51	0.608	1.24 (0.55–2.80)
Presence of isolated brachiocephalic curvature	0.95	0.41	2.31	0.021*	2.59 (1.16–5.80)
Presence of dual subclavian-brachiocephalic tortuosity	1.94	0.46	4.25	< 0.001*	6.98 (2.85–17.07)
Type of aortic arch					
Type I(Ref)					
Type II	0.42	0.34	1.24	0.216	1.53 (0.78–2.98)
Type III	0.13	0.39	0.32	0.748	1.14 (0.52–2.46)
Presence of bovine anatomy	−0.65	0.46	−1.41	0.160	0.52 (0.21–1.29)
Operators					
Neurointerventional physician 1(Ref)					
Neurointerventional physician 2	−0.09	0.30	−0.32	0.751	0.91 (0.51–1.62)

*OR *Odds Ratio, *S.E* Standard Error, *CI *Confidence Interval, *BMI *Body Mass Index

*Statistically significant

In multivariable analysis across three adjustment models, dual subclavian-brachiocephalic tortuosity remained the strongest predictor (Normal vs. Presence of dual subclavian-brachiocephalic tortuosity: OR 4.51–4.77, *p* ≤ 0.001), followed by BMI (OR 1.13–1.15, *p* ≤ 0.009), smoking (OR = 1.96–2.80, *p* ≤ 0.037) and age(OR = 1.04–1.05, *p* ≤ 0.005) (Table 4).

**Table 4:** Multivariable ordinal logistic regression analysis of risk factors across different adjustment models

Variables	Model1^a^		Model2^b^		Model3^c^	
	OR (95%CI)	*P*-value	OR (95%CI)	*P*-value	OR (95%CI)	*P*-value
Demographics						
Age	1.04 (1.01–1.08)	0.005*	1.05(1.02–1.07)	0.001*	1.05(1.02–1.08)	0.001*
Gender (Male)	1.40(0.61–3.18)	0.427	—	—	—	—
BMI	1.15(1.05–1.27)	0.004*	1.13(1.03–1.24)	0.009*	1.14(1.04–1.25)​	0.005*
Medical history						
Hypertension	1.31(0.65–2.67)	0.450	1.34(0.68–2.67)	0.399	—	—
Hyperhomocysteinemia	0.70(0.28–1.75)	0.444	—	—	—	—
Hyperlipidemia	0.90(0.48–1.71)	0.760	—	—	—	—
Diabetes	0.91(0.46–1.79)	0.790	—	—	—	—
Smoking	2.80(1.27–6.18)	0.011*	2.02(1.07–3.82)	0.030*	1.96(1.04–3.70)	0.037*
Anatomical Features						
Presence of radial artery curve	2.11(0.71–6.31)	0.196	2.33(0.79–6.84)	0.124	2.49(0.85–7.27)	0.095
Presence of brachial-axillary artery curve	1.86(0.76–4.56)	0.172	1.92(0.81–4.56)	0.139	1.91(0.80–4.52)	0.144
Right subclavian-brachiocephalic anatomy						
Normal(Ref)	1.00	—	1.00	—	1.00	—
Presence of single subclavian innominate curve	1.10(0.45–2.68)	0.912	1.05(0.44–2.51)	0.912	1.12(0.47–2.67)	0.792
Presence of isolated brachiocephalic curvature	1.48(0.59–3.74)	0.426	1.42(0.60–3.41)	0.426	1.35(0.57–3.20)	0.500
Presence of dual subclavian-brachiocephalic tortuosity	4.51(1.71–11.86)	​0.001*	4.77(1.87–12.18)	0.001*	4.70(1.85–11.92)	0.001*
Type of Aortic Arch						
Type I(Ref)	1.00	—	—	—	—	—
Type II	1.74(0.80–3.78)	0.162	—	—	—	—
Type III	0.97(0.36–2.62)	0.950	—	—	—	—
Presence of Bovine anatomy	0.57(0.20–1.57)	0.274	0.60(0.23–1.56)	0.293	—	—
Operators						
Neurointerventional physician 1(Ref)	1.00	—	—	—	—	—
Neurointerventional physician 2	1.22(0.64–2.31)	0.541	—	—	—	—

*OR *Odds Ratio, *CI *Confidence Interval, *BMI *Body Mass Index

^a^Full adjustment

^b^ P<0.2 screening

^c^ P<0.1 screening

*Statistically significant

### Mechanistic analysis of procedural determinants mediating total fluoroscopy time

To explore the potential mechanisms underlying the association between dual subclavian-brachiocephalic tortuosity and procedural complexity, we conducted a mediation analysis to investigate whether different procedural determinants mediated this relationship.

We developed a mediation model in which the presence of dual subclavian-brachiocephalic tortuosity served as the independent variable, total fluoroscopy time as the outcome, and procedural time metrics as mediators. Both SC-LSA and SC-LCCA demonstrated statistically significant mediating effects in the primary mediation model, with results remaining robust in covariate-adjusted sensitivity analyses. Conversely, procedural metrics including PTPC, CET, SC-RSA, and SC-RCCA showed non-significant mediation effects across all models, as evidenced by their 95% confidence intervals for the indirect effects (ab) encompassing zero. (Supplementary Fig. 3)

**Fig. 2:**
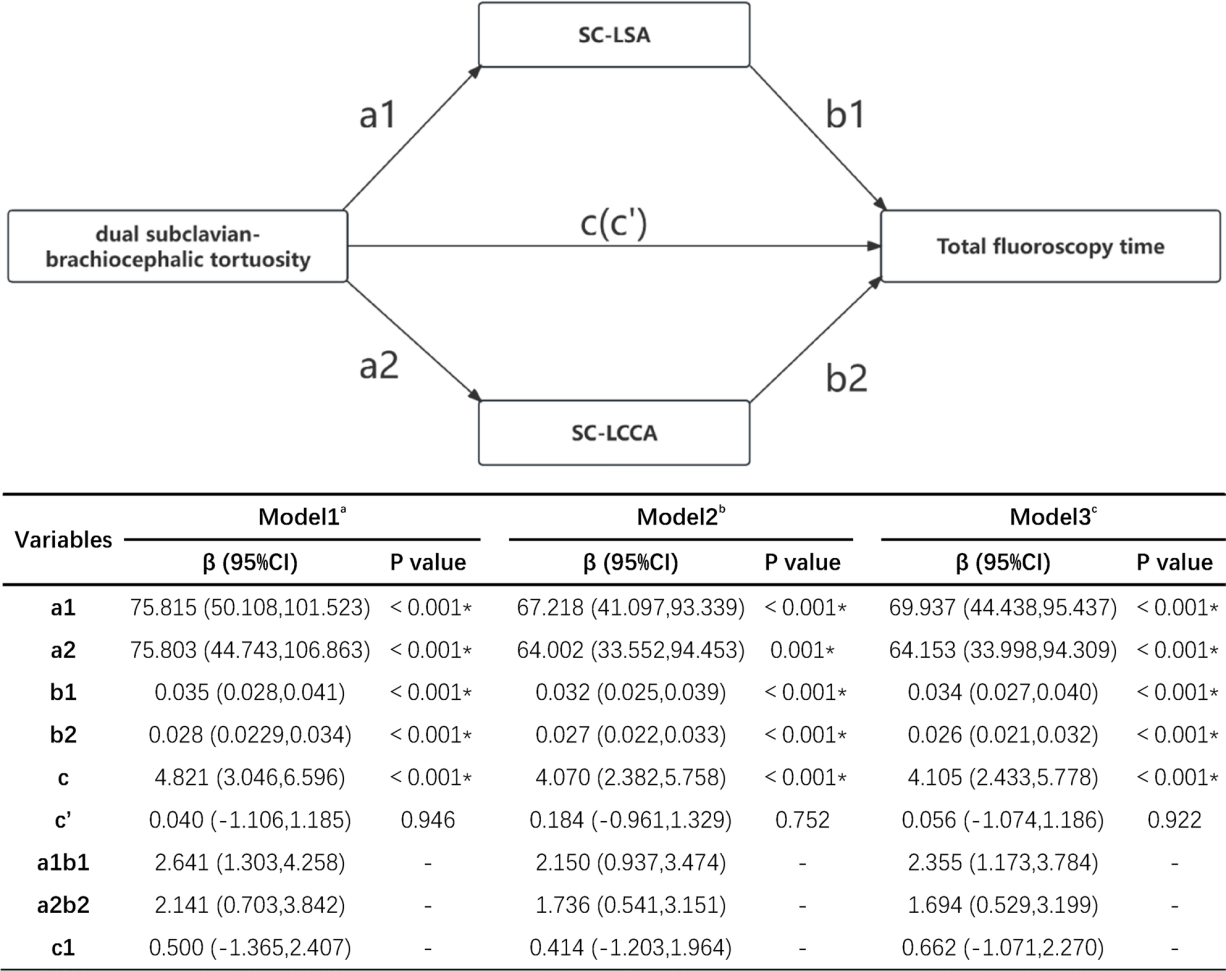
Parallel mediation analysis of procedural time metrics in the association between dual subclavian-innominate artery tortuosity and total fluoroscopy time. The mediation model is valid only if the parameters a1, b1, a2, b2 and c are simultaneously statistically significant with 95%CIs for a1b1 and a2b2 excluding zero. SC-LSA: superselective catheterization of the left subclavian artery; SC-LCCA: superselective cannulation of the left common carotid artery; CI: Confidence Interval. a: including dual subclavian-brachiocephalic tortuosity. b: including dual subclavian-brachiocephalic tortuosity and all demographics, medical history, anatomical features and Operators. c: including dual subclavian-brachiocephalic tortuosity, Age, BMI and Operators. *Statistically significant:

Further parallel mediation analysis incorporating both SC-LSA and SC-LCCA revealed that these metrics jointly mediated the effect of dual subclavian-brachiocephalic tortuosity on total fluoroscopy time. Path coefficients a1 (effect on SC-LSA), a2 (effect on SC-LCCA), b1 (SC-LSA→outcome), b2 (SC-LCCA→outcome), and c (total effect) were statistically significant across all models, with 95% confidence intervals for a1b1 and a2b2 excluding zero. The direct effect (c’) became non-significant after controlling for SC-LSA and SC-LCCA across all models, indicating complete mediation. The total indirect effect (a1b1 + a2b2) accounted for 95.5%−99.2% of the total effect (c), with the contribution ratio of SC-LSA to SC-LCCA (a1b1/a2b2) ranging from 1.2:1 to 1.4:1. However, the 95% confidence intervals for the contrast effect (c1) between SC-LSA and SC-LCCA included zero in all models, suggesting no statistically significant difference in their mediating effects (Fig. 2).

## Discussoin

This study establishes dual subclavian-brachiocephalic tortuosity as a potent anatomical determinant of procedural inefficiency in TRA cerebral angiography. Patients exhibiting this specific vascular configuration faced almost a 5-fold increased risk of prolonged total fluoroscopy time, surpassing the impact of conventional risk factors like age and BMI. Crucially, the resultant “3-shaped” catheter deformation (Fig. 1D) predominantly impedes superselective catheterization of left-sided arch vessels (LSA and LCCA), accounting for > 95% of the total effect on fluoroscopy time through parallel mediation pathways. These findings illuminate a previously underappreciated anatomical barrier in neurointerventional radial access and provide actionable insights for optimizing procedural strategy.

While TRA has transformed cardiovascular interventions by reducing puncture-site complications, improving patient comfort, and lowering mortality rates [12–14], its adoption in neuroendovascular procedures remains limited [6, 7]. Despite confirmed safety and feasibility of transradial cerebral angiography [2, 15–18], prolonged fluoroscopy times in specific subsets remain a key barrier to widespread adoption [10, 11, 19]. Our data corroborates this heterogeneity: while 72.9% of procedures (Easy/Moderate groups) concluded within 10 min, 27.1% required > 10 min (Difficult/Extremely difficult), with 9.4% exceeding 15 min. This bifurcation aligns with Kenawy et al.’s observation [6] of variable efficiency in TRA angiography, but our anatomical stratification reveals why such disparities exist.

**Fig. 3:**
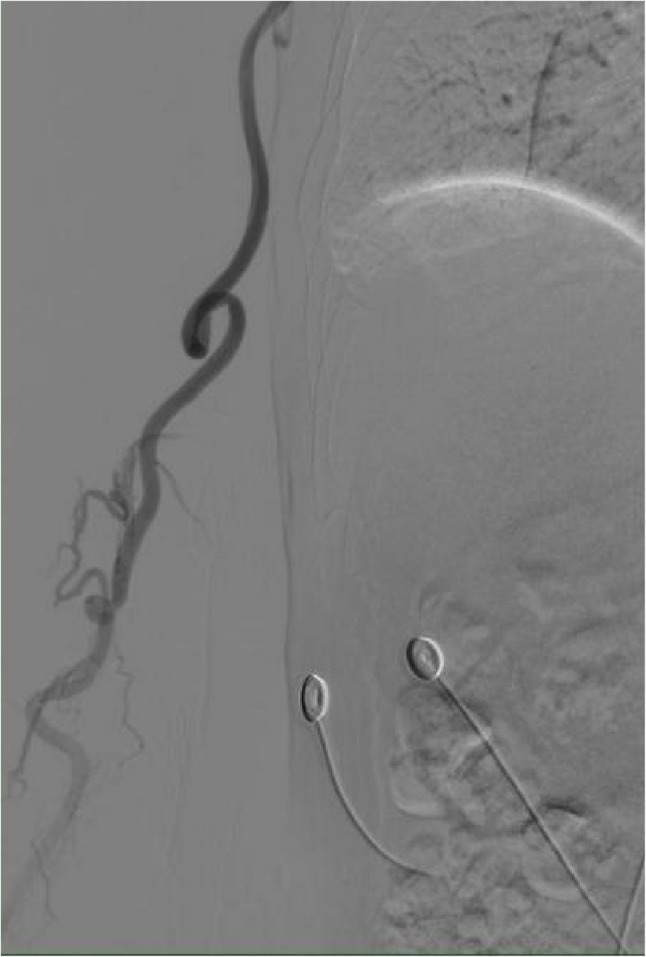
Presence of Radial Artery Multiple Kinks and Loops. As shown in the figure, this is a retrograde angiogram via the radial artery sheath, where multiple tortuosities and loops of the right radial artery are visible. The guidewire could not smoothly pass through all the tortuous anatomical structures, leading to a failure of radial artery access for angiography. After switching to femoral artery puncture and sheath insertion, the angiography was successfully completed.

**Fig. 4:**
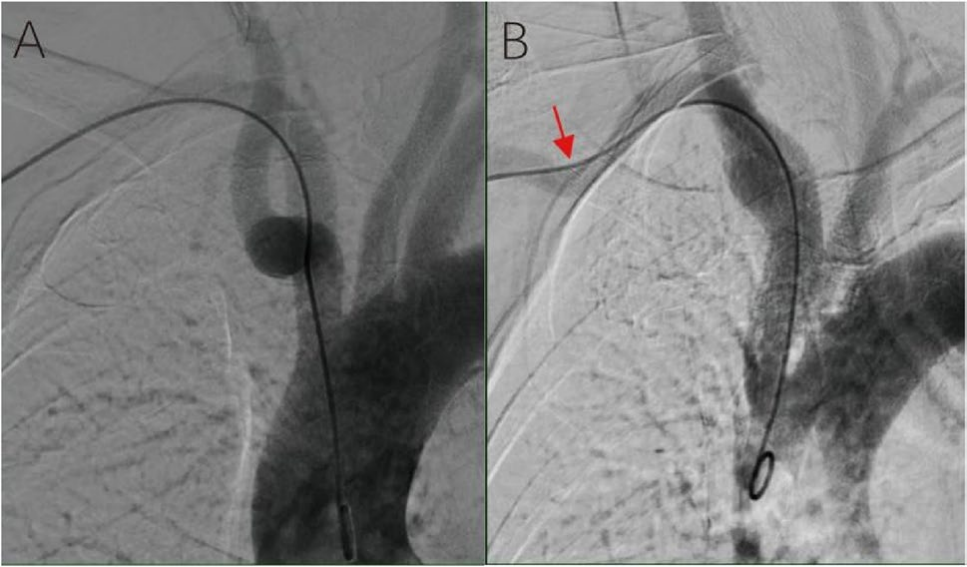
Morphology of Brachial-axillary Artery. **A** Normal brachial-axillary arterial. **B** Presence of brachial-axillary arterial curve (red arrow）

The fundamental challenge lies in navigating the complex pathway from the radial artery to the aortic arch. Unlike transfemoral access, TRA requires traversal of smaller-caliber vessels before engaging the aortic arch. While radial artery tortuosity may preclude vascular access entirely (Fig. 3, excluded in our cohort), and brachial-axillary arterial curve (Fig. 4B) increases radial resistance during catheter navigation yet remains readily overcome with guidewire support, subclavian-brachiocephalic morphology proves critically disruptive. Normal or singly tortuous segments rarely induce significant catheter deformation (Fig. 1A–C), but dual subclavian-brachiocephalic tortuosity creates a fixed “3-shaped” catheter conformation that simultaneously compromises axial pushability and rotational torque transmission—a mechanical vulnerability exacerbated during superselective maneuvers requiring precise catheter torque. These findings directly concur with the researches of Khan NR et al. [4] and Brunet, MC et al. [7].

Our mediation analysis provides mechanistic clarity: dual tortuosity primarily impacts LSA and LCCA catheterization. This selective effect arises from the compounded biomechanical disadvantage when manipulating catheters from the right radial access toward left-sided vessels. The Simmons II catheter must traverse the aortic arch’s apex and navigate caudally toward left subclavian/carotid origins—a maneuver demanding efficient torque transfer through the catheter shaft. The “3-shaped” deformation in the proximal pathway (Fig. 1D) dissipates rotational forces, necessitating repeated adjustments under live fluoroscopy. Conversely, RCCA superselective catheterization involves directing the catheter tip toward the brachiocephalic trunk. Gentle contrast injection coupled with gentle rotation and cephalad retraction guides the catheter tip into position along the RCCA(Supplementary video 1). Operators with moderate experience typically complete this maneuver without roadmap guidance or guidewire assistance, generally requiring minimal time. Similarly, RSA catheterization via TRA ​involves withdrawing the catheter from the RCCA while administering gentle contrast injection to position the tip in the proximal RSA or at the origin of the right vertebral artery (Supplementary video 2). Consequently, this difficulty gradient manifested in significantly prolonged median times for SC-LSA (173.50s) and SC-LCCA (180.50s) in the Extremely Difficult group, eclipsing the Easy group’s 24.00s and 17.00s. In stark contrast, RCCA/RSA procedures maintained efficiency across cohorts at 30.00s(21.00–48.75 s)/33.00s(22.00–45.75 s) regardless of tortuosity severity. This differential difficulty explains the disproportionate radiation burden between extremely difficult group and easy group (fDAP95.38µGy·m² vs. 19.50µGy·m²), elevating risks of skin injury, contrast nephropathy, and catheter fatigue complications [7, 20].

Notably, other anatomical factors (aortic arch type, bovine variant) showed no significant association with difficulty, contradicting reports in literature [4, 21]. Our cohort exclusively included patients undergoing targeted catheterization of supra-aortic trunks (LSA, LCCA, RSA, RCCA) via TRA, excluding those requiring branch vessel selection (e.g., internal carotid/vertebral artery). This selective design ensured procedural consistency, enabled quantifiable time segmentation, and critically eliminated outcome bias from extended fluoroscopy during additional catheter and guidewire manipulations. However, aortic arch type II-III may decrease the provision of proper proximal support, which causes kickbacks of the catheter during attempted guidewire navigation of the left internal carotid [21]. Meanwhile, superselective catheterization of the left vertebral artery often necessitates advanced techniques such as dual wire anchoring technique to overcome arch-induced instability [4]. Consequently, the selective design reconciles our null findings with prior studies reporting anatomical influences, demonstrating that arch effects manifest predominantly during branch catheterization. In our study, bovine configuration showed no correlation with total fluoroscopy time or procedural difficulty. This finding does not contradict the observations by Nickalus et al., whose research demonstrated that bovine anatomy was associated with fewer catheters used but exhibited no significant reduction in operator-rated difficulty or fluoroscopy time per vessel [4]. This pattern suggests bovine arches primarily facilitate selective catheterization of the LCCA, while exerting minimal impact on radiation exposure metrics. However, both our study and Nickalus et al.‘s work share a critical limitation: bovine arch cohorts were relatively small (our cohort: *n* = 18; Nickalus et al.: *n* = 12). Definitive validation through expanded samples remains necessary to establish statistical robustness.

This study had several limitations. First, the retrospective single-center design with limited cohort size inherently constrained statistical power and generalizability, while low representation of critical anatomical variants (e.g., bovine arch) may obscure their clinical relevance. Second, the exclusion of crossover cases (*n* = 17) due to radial access failure may underestimate the true clinical burden of right radial artery tortuosity, rendering our reported odds ratio for dual subclavian-brachiocephalic tortuosity a conservative clinical estimate. Third, this study employed a qualitative approach for anatomical assessment, utilizing intraprocedural angiography and catheter torque characteristics instead of quantitative measurements such as angulation, segmental distances or luminal diameters. While efficient, this method sacrificed metric precision, a limitation compounded by the non-routine application of preprocedural CTA for vessel reconstruction in a subset of patients. Finally, by standardizing the procedure with 5Fr simmons II catheter to minimize bias, this study specifically investigated the impact of vascular tortuosity on angiography difficulty. Consequently, this design choice did not allow for the evaluation of torque control differences between catheter designs or materials, which is an important direction for future investigation.

## Conclusions

Dual subclavian-brachiocephalic tortuosity is a critical yet remediable obstacle to efficient TRA cerebral angiography. Its propensity to induce “3-shaped” catheter deformation severely impedes left-sided vessel selection, substantially prolonging fluoroscopy time and radiation exposure. Recognition of this anatomical variant enables risk-stratified patient selection and targeted technical refinements, ultimately advancing TRA as a viable strategy for neurointerventional diagnostics. Prospective validation of these findings and device innovations tailored to neurovascular anatomy represent essential next steps.

## Supplementary Information

Supplementary Video 2.

Supplementary Video 1.

Supplementary Material(table and figures).

## Data Availability

The data that support the findings of this study are available on request from the corresponding author. The data are not publicly available due to privacy or ethical restrictions.

## Abbreviations

TRA: Trans-Radial Access
CI: Confidence Interval
OR: Odds Ratio
SD: Standard deviation
IQR: Interquartile range
S.E: Standard Error
BMI: Body Mass Index
fDAP: Fluoroscopy dose-area product
PTPC: Placement time of pigtail catheter
CET: Catheter Exchange Time
SC-LSA: Superselective Catheterization of the Left Subclavian Artery
SC-LCCA: Superselective Cannulation of the Left Common Carotid Artery
SC-RSA: Superselective cannulation of the Right subclavian artery
SC-RCCA: Superselective cannulation of the Right Common Carotid Artery
CTA: Computed tomography angiography
