# Supplementary material for: Dual subclavian-brachiocephalic artery tortuosity and its impact on procedural efficiency in trans-radial access cerebral angiography: a retrospective cohort study
Source: BMC Neurol. 2026 Mar 10;26:255. doi: 10.1186/s12883-026-04799-4 (PMC13085326; doi:10.1186/s12883-026-04799-4)
Supplement: Supplementary file 3 — Supplementary Material(table and figures). [file 12883_2026_4799_MOESM3_ESM.pdf]

Supplementary Table. Procedural Time Metrics of Different Operators

Characteristics	Total (n=170)	Neurointerventional physician 1 (n=73)	Neurointerventional physician 2 (n=97)	*Z-value*	*P-value*
Total fluoroscopy time, min, IQR	7.27 (5.32, 10.12)	8.48 (5.85, 10.13)	6.63 (5.28, 8.50)	-1.96	0.050
PTPC, second, IQR	34.00 (29.00, 39.00)	34.00 (29.00, 39.00)	34.00 (28.00, 39.00)	-0.33	0.742
CET, second, IQR	40.00 (35.00, 46.75)	42.00 (37.00, 46.00)	39.00 (34.00, 48.00)	-1.26	0.208
SC-LSA, second, IQR	52.50 (33.25, 85.75)	60.00 (35.00, 102.00)	47.00 (32.00, 71.00)	-2.00	0.045*
SC- LCCA, second, IQR	36.50 (24.00, 70.00)	49.00 (23.00, 82.00)	35.00 (24.00, 57.00)	-1.17	0.241
SC-RCCA, second, IQR	33.00 (22.00, 45.75)	34.00 (25.00, 50.00)	32.00 (21.00, 40.00)	-1.13	0.260
SC-RSA, second, IQR	30.00 (21.00, 48.75)	30.00 (21.00, 47.00)	30.00 (21.00, 49.00)	-0.10	0.921

PTPC：placement time of pigtail catheter; CET：catheter exchange time; SC-LSA：superselective catheterization of the left subclavian artery; SC-LCCA：superselective cannulation of the left common carotid artery; SC-RSA：superselective cannulation of the right subclavian artery; SC-RCCA：superselective cannulation of the right common carotid artery; SD: standard deviation; IQR, interquartile range.

Z: Mann-Whitney test

*Statistically significant

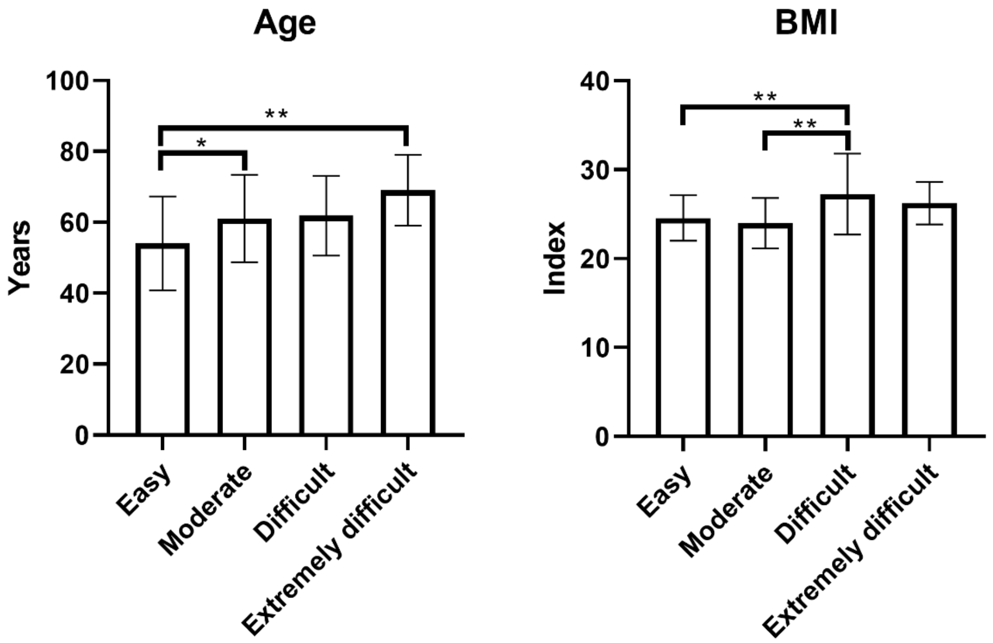

Supplementary figure 1. Intergroup comparisons of age and BMI across difficulty levels (Bonferroni-corrected analysis)

*** P＜0.05 ** P＜0.01**

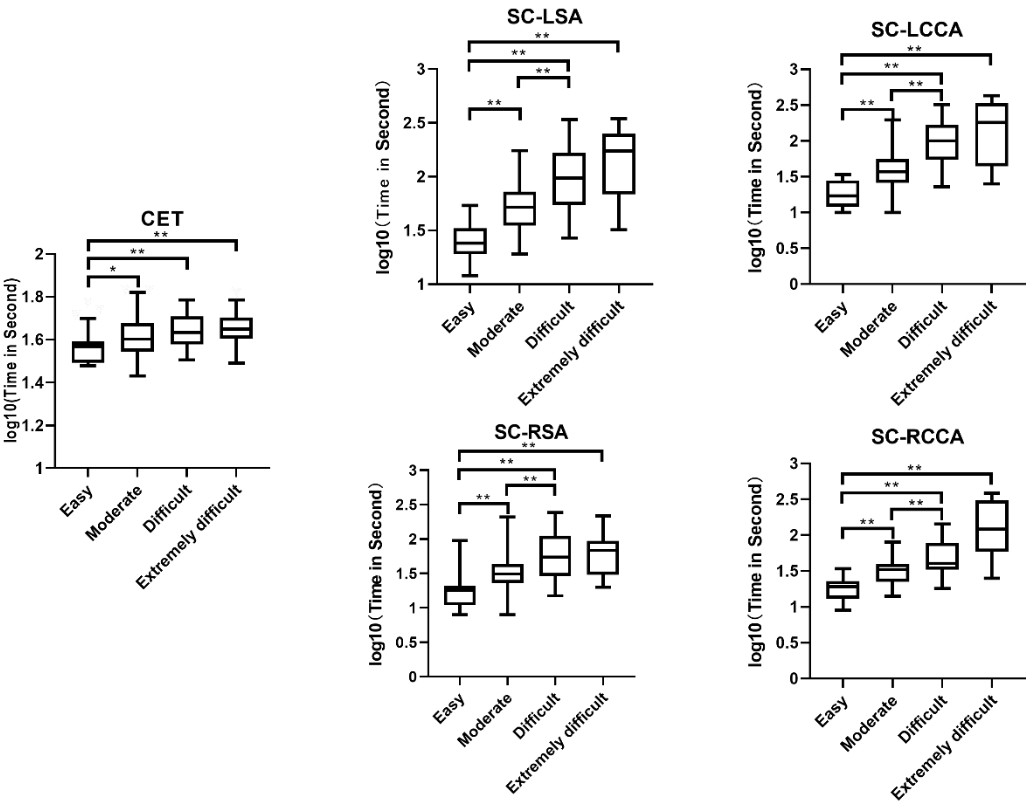

Supplementary figure 2. Intergroup comparisons of CET, SC-LSA, SC-LCCA, SC-RSA, and SC-RCCA across difficulty levels (Kruskal-Wallis H test with Dunn's correction for multiple comparisons)

*** P＜0.05 ** P＜0.01**

Supplementary figure 3 The interventional procedures mediate the effect of dual subclavian-innominate artery tortuosity on total fluoroscopy time.

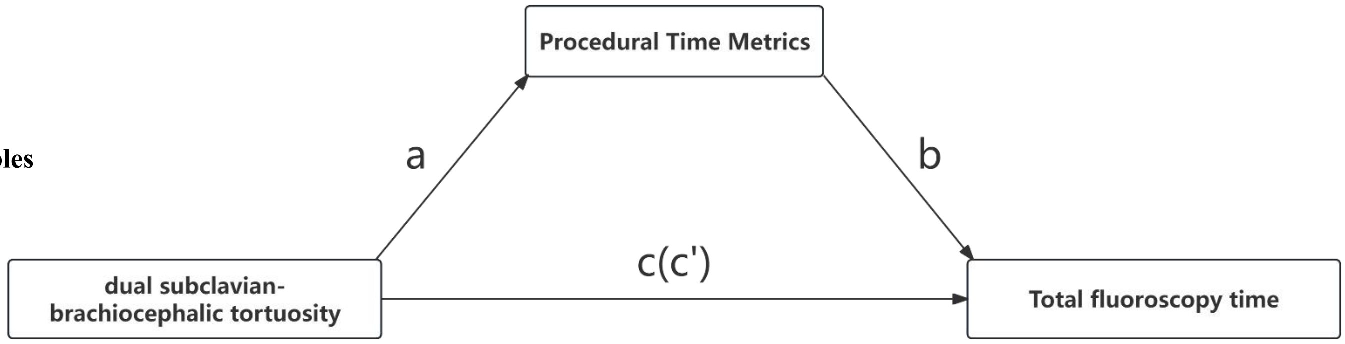

Variables	Model1		Model2		Model3	
	β (95%CI)	P-value	β(95%CI)	P-value	β(95%CI)	P-value
**PTPC**						
a	1.031 (-2.635,4.698)	0.579	1.184 (-2.561,4.929)	0.624	0.792 (-2.979,4.563)	0.679
b	0.025 (-0.048,0.100)	0.490	0.039 (-0.033,0.110)	0.287	0.024 (-0.045,0.092)	0.497
c	4.821 (3.046,6.596)	< 0.001*	4.070 (2.382,5.758)	< 0.001*	4.105 (2.433,5.778)	< 0.001*
c'	4.794 (3.015,6.574)	< 0.001*	4.024 (2.334,5.714)	< 0.001*	4.087 (2.411,5.763)	< 0.001*
ab	0.027(-0.136,0.298)	-	0.046（-0.141，0.370）	-	0.187（-0.125，0.271）	-
**CET**						
a	2.466 (-1.291,6.224)	0.197	1.245 (-2.544,5.035)	0.517	1.437 (-2.299,5.174)	0.449
b	0.121 (0.051,0.191)	< 0.001*	0.088 (0.018,0.157)	0.006*	0.076 (0.081,0.144)	0.027*
c	4.821 (3.046,6.596)	< 0.001*	4.070 (2.382,5.758)	< 0.001*	4.105 (2.433,5.778)	< 0.001*
c'	4.523 (2.793,6.253)	< 0.001*	3.961 (2.298,5.624)	< 0.001*	3.996 (2.340,5.652)	< 0.001*
ab	0.298（-0.176，0.936）	-	0.109(-0.208,0.514)	-	0.109(-0.167,0.524)	-
**SC-LSA**						
a	75.815 (50.108,101.523)	< 0.001*	67.218 (41.097,93.339)	< 0.001*	69.937 (44.438,95.437)	< 0.001*
b	0.047 (0.039,0.055)	< 0.001*	0.041 (0.033,0.049)	< 0.001*	0.043 (0.035,0.050)	< 0.001*
c	4.821 (3.046,6.596)	< 0.001*	4.070 (2.382,5.758)	< 0.001*	4.105 (2.433,5.778)	< 0.001*
c'	1.280 (-0.158,2.718)	0.081	1.294 (-0.113,2.701)	0.071	1.120 (-0.262,2.502)	0.111
ab	3.543 (1.740,5.600)	-	2.776 (1.188,4.526)	-	2.985 (1.433,4.830)	-
**SC-LCCA**						
a	75.80 (44.97,106.64)	< 0.001*	64.002 (33.552,94.453)	0.001*	64.153 (33.998,94.309)	< 0.001*
b	0.04 (0.03,0.04)	< 0.001*	0.035 (0.028,0.042)	< 0.001*	0.035 (0.028,0.041)	< 0.001*
c	4.821 (3.046,6.596)	< 0.001*	4.070 (2.382,5.758)	< 0.001*	4.105 (2.433,5.778)	< 0.001*
c'	1.92 (0.52,3.32)	0.008*	1.817 (0.437,3.196)	0.010*	1.885 (0.506,3.263)	0.007*
ab	2.901 (0.998,5.177)	-	2.253 (0.652,4.175)	-	2.221 (0.660,4.235)	-
**SC-RSA**						
a	18.690 (-0.159,37.538)	0.052	18.314 (-1.334,37.962)	0.068	16.007 (-3.107,35.121)	0.100
b	0.041 (0.028,0.054)	< 0.001*	0.035 (0.022,0.047)	< 0.001*	0.035 (0.023,0.048)	< 0.001*
c	4.821 (3.046,6.596)	< 0.001*	4.070 (2.382,5.758)	< 0.001*	4.105 (2.433,5.778)	< 0.001*
c'	4.059 (2.436,5.683)	< 0.001*	3.430 (1.866,4.995)	< 0.001*	3.540 (1.993,5.087)	< 0.001*

Variables	Model1		Model2		Model3	
	β (95%CI)	P-value	β(95%CI)	P-value	β(95%CI)	P-value
ab	0.762（-0.090，1.756）	-	0.640（-0.141，1.47）	-	0.566（-0.192，1.434）	-
**SC-RCCA**						
a	32.963 (8.421,57.685)	0.009*	28.917 (3.894,53.940)	0.024*	27.927 (3.194,52.660)	0.039*
b	0.045 (0.036,0.054)	< 0.001*	0.040 (0.031,0.049)	< 0.001*	0.042 (0.033,0.050)	< 0.001*
c	4.821 (3.046,6.596)	< 0.001*	4.070 (2.382,5.758)	< 0.001*	4.105 (2.433,5.778)	< 0.001*
c’	3.338 (1.922,4.755)	< 0.001*	2.915 (1.527,4.303)	0.001*	2.947 (1.603,4.292)	< 0.001*
ab	1.483(-0.058,3.196)	-	1.143(-1.194,2.554)	-	1.158(-0.285,2.689)	-

PTPC：Placement time of pigtail catheter;CET：Catheter exchange Time;SC-LSA：Superselective catheterization of the left subclavian artery;SC-LCCA：Superselective cannulation of the left common carotid artery;SC-RSA：Superselective cannulation of the right subclavian artery;SC-RCCA：Superselective cannulation of the right common carotid artery;CI: Confidence Interval；Model1：Adjust none；Model2：Adjust full factors；Model3：Adjust Age, BMI and Operators

*Statistically significant
